# Electroconductive Composites from Polystyrene Block Copolymers and Cu–Alumina Filler

**DOI:** 10.3390/ma9120989

**Published:** 2016-12-07

**Authors:** QuratulAin Nadeem, Tasneem Fatima, Pepijn Prinsen, Aziz ur Rehman, Rohama Gill, Rashid Mahmood, Rafael Luque

**Affiliations:** 1 Department of Environmental Sciences, Fatima Jinnah Women University, Rawalpindi 46000, Pakistan; quratulainnadeem@hotmail.com (Q.N.); tasnem009@gmail.com (T.F.); 2 Departamento de Universidad de Córdoba, Edificio Marie Curie, Ctra Nnal IV-A, Km396, E14014 Córdoba, Spain; pepijnprinsen33@hotmail.com (P.P.); q62alsor@uco.es (R.L.); 3 Department of Chemistry, the Islamia University of Bahawalpur, Bahawalpur 63000, Pakistan; draziz@iub.edu.pk; 4 Department of Chemistry, University of Azad Jammu and Kashmir Chehla Campus Muzaffarabad, Muzaffarabad 13100, Pakistan; rashid_meh786@yahoo.com

**Keywords:** copolymers, composites, morphology, mechanical properties, thermal properties

## Abstract

Technological advancements and development of new materials may lead to the manufacture of sustainable energy-conducting devices used in the energy sector. This research attempts to fabricate novel electroconductive and mechanically stable nanocomposites via an electroless deposition (ELD) technique using electrically insulating materials. Metallic Cu is coated onto Al_2_O_3_ by ELD, and the prepared filler is then integrated (2–14 wt %) into a matrix of polystyrene-block-poly(ethylene-ran-butylene)-block-polystyrene-graft-maleic anhydride (PS-*b*-(PE-*r*-B)-*b*-PS-*g*-MA). Considerable variations in composite phases with filler inclusion exist. The Cu crystallite growth onto Al_2_O_3_ was evaluated by X-ray diffraction (XRD) analysis and energy dispersive spectrometry (EDS). Scanning electron microscopy (SEM) depicts a uniform Cu coating on Al_2_O_3_, while homogeneous filler dispersion is exhibited in the case of composites. The electrical behavior of composites is enhanced drastically (7.7 × 10^−5^ S/cm) upon incorporation of Cu–Al_2_O_3_ into an insulating polymer matrix (4.4 × 10^−16^ S/cm). Moreover, mechanical (Young’s modulus, tensile strength and % elongation at break) and thermal (thermogravimetric analysis (TGA), derivative thermogravimetry (DTG), and differential scanning calorimetry (DSC)) properties of the nanocomposites also improve substantially. These composites are likely to meet the demands of modern high-strength electroconductive devices.

## 1. Introduction

Technological advances highly depend on the development of a wide diversity of new materials. Conductive polymer composites (CPCs) have an array of applications in various industries, among them the electronic industry, which made revolutionary developments both in manufacturing and recycling. Electrostatic discharge (ESD) and electromagnetic interference (EMI) are phenomena that affect the economy of the electronic industry. They can arise during manufacturing, packing, conveyance, and working. Thus, the use of appropriate EMI-shielding materials to reduce electric energy losses is essential [1]. The ever-growing electronic waste (e-waste) is now posing devastating impact on the environment due to its accumulation. One way to reduce this accumulation is to increase the life span of electronics to protect them from the detrimental effects of EMI and ESD. Design and application of CPCs as advanced materials have been shown to expand the shelf life of electronics, which may ultimately reduce the production of e-waste [2]. Although, production of various classes of conducting polymer nanocomposites on a commercial scale is growing at a rapid pace, yet metal-filled CPCs exhibit poor mechanical properties and are no longer preferred by modern industries due to their high cost. To provide exceptional electrical properties without compromising the mechanical behavior, researchers switched their focus towards metal-coating techniques such as electrodeposition, chemical vapor deposition, physical vapor deposition, electrospinning, and others. Among them, electroless deposition (ELD) is a novel metal deposition technique. The ELD-plating technique is able to incorporate desired properties of a metallic coating irrespective of the substrate geometry and at low temperature. This coating technique is redox-sensitive, as the internal current is supplied by the oxidation of a reducing agent [3,4], thus uniform plating can also be carried out inside the holes, recesses, and non-line-of-sight surfaces [5,6,7]. A variety of metals—such as Ag, Cu, Au, and Ni (in order of decreasing conductivity)—have been coated on different substrates via ELD to fabricate electrically conductive materials like conductive plates, wires, rods, and powders for various electronic applications [8,9,10].

The novelty of the present research work lies in the preparation of electrically conductive Cu-coated alumina powder via ELD, which was then used, for the first time, as filler in a matrix of polystyrene-block-poly(ethylene-ran-butylene)-block-polystyrene-graft-maleic anhydride (PS-*b*-(PE-*r*-B)-*b*-PS-*g*-MA). The selected polymer matrix is electrically insulating and offers the characteristics of vulcanized rubber without going through the process of vulcanization. Also, the presence of styrene maleic anhydride (SMA) segments in the copolymer elevates the glass transition temperature (T_g_). The appropriate interfacial properties of the matrix [11,12] makes it suitable for the preparation of blends and composites. The limitations of the selected copolymer are its low strength and stiffness [13]. Alumina is used in numerous applications in various fields due to its excellent mechanical properties, anticorrosivity, wear resistance, and hardness. The presence of Cu in the ELD-deposited metal-ceramic filler enhances the electrical conductivity and the incorporation of Al_2_O_3_ increases the mechanical strength, compensating for the low strength and stiffness of the copolymer. The resultant concoction may improve the durability of advanced material applications, such as EMI- and ESD-shielding materials [8], heat sinks for microelectronics [14], sensors for biomedical usage [9], and so on.

## 2. Experimental Section

This research work focused on the synthesis and characterization of conductive composites by adding a conductive ceramic filler, coated with a metal through ELD technique, in a nonconducting polymer.

### 2.1. Materials

The following analytical-grade chemicals were used: Fluka Chemika (Buchs, Switzerland, aluminium oxide (Al_2_O_3_)), Riedel-de Haen (Seelze, Germany, nitric acid (HNO_3_ 37%), copper sulphate pentahydrate (CuSO_4_·5H_2_O), and potassium sodium tartrate (KNaC_4_H_4_O_6_·4H_2_O)), Merck (Darmstadt, Germany, hydrofluoric acid (HF)), Scharlau (Barcelona, Spain, sodium hydroxide (NaOH)), Sigma Aldrich (Buchs, Switzerland, polyethylene glycol (C_2n_H_4n+2_O_n+1_), thiourea (CH_4_N_2_S), palladium chloride (PdCl_2_), stannous chloride (SnCl_2_·2H_2_O), ethylenediaminetetraacetic acid disodium salt (C_10_H_14_N_2_Na_2_O_8_), formaldehyde solution (HCHO), dimethylamine borane (C_4_H_10_BN), boric acid (H_3_BO_3_), chloroform (CHCl_3_), and polystyrene-block-poly(ethylene-ran-butylene)-block-polystyrene-graft-maleic anhydride (PS-*b*-(PE-*r*-B)-*b*-PS-*g*-MA)).

### 2.2. Preparation of Conductive Filler

Electroless deposition (ELD) method was employed for the preparation of conductive filler. Copper (Cu) deposition onto Al_2_O_3_ substrate was accomplished after successive substrate pretreatment steps.

#### 2.2.1. Pretreatment of Al_2_O_3_

Pretreatment of Al_2_O_3_ was done before the deposition step, followed by surface cleaning and surface activation. To avoid tedious filtration steps, Al_2_O_3_ was packed in commercially available silk cloth (160 mesh) and was dipped in subsequent solutions rather than dispersion in solution, which may also increase the reaction time. First, the Al_2_O_3_ substrate was dipped in concentrated HNO_3_ (2 min) to remove oil and dirt. Acid-cleaned Al_2_O_3_ was dipped in catalytic activator solution containing 0.03 mmol of PdCl_2_ and 0.246 mmol of SnCl_2_ in 40 mL of concentrated HCl (14 min). After activation, the substrate was introduced to a reduction bath made of 4.74 mmol of (C_4_H_10_BN) and 4.52 mmol of (H_3_BO_3_) in a sufficient quantity of distilled water (7 min). Each step of pretreatment was followed by 1 min rinsing in distilled water. Pretreated Al_2_O_3_ was then used for ELD of Cu.

#### 2.2.2. Cu Coating on Pretreated Al_2_O_3_

Pretreated Al_2_O_3_ was dipped in an electroless plating bath (Table 1). After deposition, Cu-deposited Al_2_O_3_ was rinsed with distilled water and oven-dried for 3–4 h at 40 °C. The prepared Cu–Al_2_O_3_ powder was used further as conductive filler for insulating polymer matrix.

### 2.3. Synthesis of Conductive Composites

Conductive polymer composites (CPCs) were prepared by incorporating Cu–Al_2_O_3_ filler with varied content (2, 4, 6, 8, 10, 12, and 14 wt %) in PS-*b*-(PE-*r*-B)-*b*-PS-*g*-MA polymer matrix. PS-*b*-(PE-*r*-B)-*b*-PS-*g*-MA was dissolved in 30 mL chloroform followed by addition of the filler. The polymer–filler solution was stirred for 1–2 h at 750 rpm, poured into a Petri dish for film casting, and then detached from the mold after solvent evaporation. The prepared composite films were utilized for characterization. To attain accuracy in performance and results, samples were prepared in triplicates and the mean values were reported after characterization.

### 2.4. Instrumentation and Characterization

#### 2.4.1. X-ray Diffraction (XRD) Analysis

PANalytical X-ray diffractometer (XPERT-PRO) (Düsseldorf, Germany) was used for XRD analysis of pristine Al_2_O_3_, Cu–Al_2_O_3_, PS-*b*-(PE-*r*-B)-*b*-PS-*g*-MA, and Cu–Al_2_O_3_/PS-*b*-(PE-*r*-B)-*b*-PS-*g*-MA composites. As Cu is the anodic material, X-rays of wavelength 1.540598 Å (Cu-Kα) were used for analysis. The 2*θ* data were analyzed with 0.05° scan step size, scan range 5°–70° (1 s) at 40 kV voltage and 30 mA beam current. The *d*-spacing and average crystallite size of Cu and Cu–Al_2_O_3_ particles were calculated by Bragg’s and Scherrer’s equation, respectively.

#### 2.4.2. Morphological Analysis

The surface morphologies of pristine Al_2_O_3_, Cu-coated Al_2_O_3_, host polymer, and its respective composites were analyzed with SEM, obtained by a HT-Phys-UAJK microscope equipped with a secondary electron (SE) detector at 25 kV accelerating voltage. Fractured surfaces of composites were also examined by MIRA3 TESCAN (Nova 400 Nano, Salem, OR, USA) (SE detector at an accelerating voltage of 10 kV) to analyze the dispersion of filler in polymer matrix.

#### 2.4.3. Energy Dispersive Spectrometry (EDS) Analysis

Elemental composition and atomic weight % of Cu coated Al_2_O_3_, host polymer and its respective composites were investigated by using a JSM6490LV (JEOL) microscope (Tokyo, Japan). The instrument was equipped with QUANTAX EDS XFlash detector 4010-Bruker (Billerica, MA, USA) at an accelerating voltage of 20 kV.

#### 2.4.4. Analysis of Surface/Volume Resistivity and Electrical Conductivity

The surface resistivity (Ω/□) and volume resistivity (Ω·cm) of composites were measured by the 4-probe method using a high-resistance meter by applying the ASTM D-257 test method [15] at room temperature. A 500 V direct current field was applied through electrodes made up of tungsten carbide. Since electrical conductivity is inversely proportional to volume resistivity, electrical conductivities (S/cm) were calculated as the inverse of the volume resistivities (Ω·cm).

#### 2.4.5. Analysis of Mechanical Properties

The mechanical features of composites were examined by calculating the Young’s modulus (MPa), tensile strength (MPa), and % elongation at break according to the ASTM D638-02 [16] and ASTM D638-03 [17] test procedures for mechanical analysis. An Instron tester (4465UK, Norwood, MA, USA) was used at 20 ± 2 °C by subjecting samples with dimensions of 0.8–1.0 mm thickness and 6 mm× 70 mm (width × gauze length).

#### 2.4.6. Analysis of Thermal Properties

Thermogravimetric analysis (TGA) was carried out with a Perkin Elmer TGA-7 (Waltham, MA, USA) in the 50–550 °C temperature range at 20 °C/min in dynamic atmosphere (20 mL/min N_2_ flow) using a 2 mg sample. Non-isothermal conditions were used for recording thermal analytical results. A DSC 404-NETZSCH instrument was used for differential scanning calorimetry (DSC) analysis in the 20–500 °C range at 20 °C/min.

## 3. Results and Discussion

### 3.1. XRD Analysis of Pristine and Cu-Coated Al_2_O_3_ Powder and Composite Films

The prepared filler was analyzed with XRD for the determination of phase change and particle size. Scherrer’s equation is used to calculate the particle size of pristine and Cu-coated Al_2_O_3_ as expressed in Equation (1) [18]:

(1) $$D=\frac{K\lambda }{\beta \;cos\theta }$$

where *D* = crystallite size (nm); *λ* = wavelength; *K* = Scherrer’s constant; *β* = angular width (radians); and *θ* = Bragg’s angle.

Interplanar spacing between atoms within the crystallite structure is denoted by *d*-spacing. Bragg’s equation used for the determination of *d*-spacing of pristine and Cu-coated Al_2_O_3_ is given in Equation (2).

(2) 2dsinθ = n λ (n−1)

**Figure 1:**
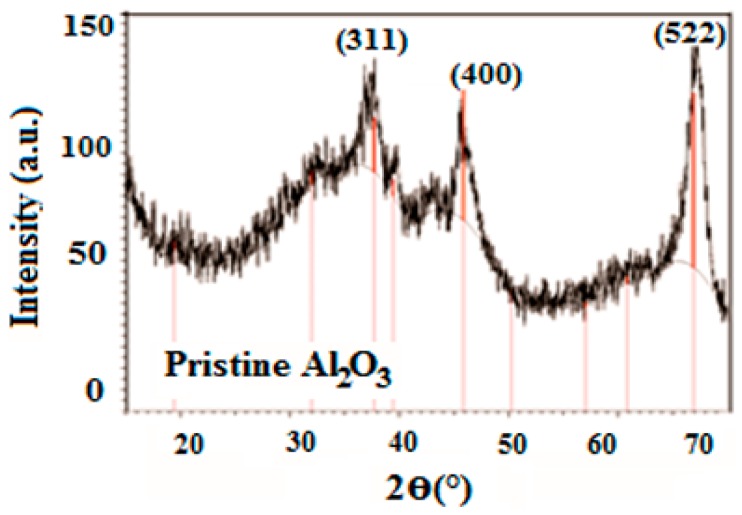
X-ray diffraction (XRD) pattern of pristine Al_2_O_3_ nanopowder.

The XRD spectrum of pristine Al_2_O_3_ powder is presented in Figure 1. The three peaks at 13.13°, 46.04°, and 67.28° (2*θ* values), correspond to the Al_2_O_3_ phase. The strongest diffraction peak is observed at 67.28° with minimum *d*-spacing 0.139 nm. At 46.04°, another peak exists corresponding to Al_2_O_3_ with *d*-spacing 0.197 nm. The average size of the cubic lattice of Al_2_O_3_ is approximately 8.8 nm.

**Figure 2:**
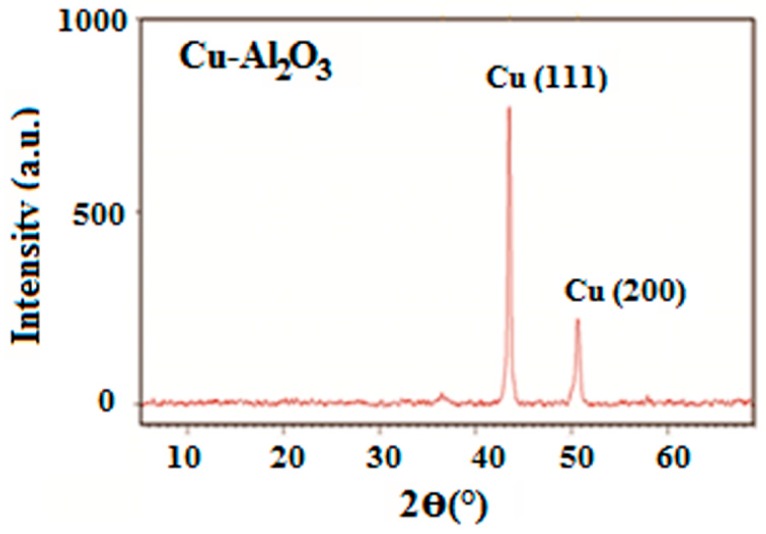
XRD pattern of Cu-coated Al_2_O_3_ nanopowder.

The XRD spectrum of Cu–Al_2_O_3_ (Figure 2) depicts the strongest peak at 43.5° and a relatively less intense peak at 50.6° corresponding to the (111) and (200) lattice planes of Cu, respectively. The strongest diffraction peak at 43.5° is characteristic of a face-centered cubic structure with *d*-spacing of 0.21 nm; this confirms deposition of crystal-structured metallic Cu on the substrate [18,19]. The relative peak intensity at 2*θ* = 67.3° clearly represents the XRD pattern of pristine Al_2_O_3_ (Figure 1), whose amount was lower in the composite material. The disturbance observed in the peak corresponding to the Al_2_O_3_ phase is due to the change in the nature of original Al_2_O_3_ after the deposition of Cu. The average crystallite size of Cu–Al_2_O_3_ was calculated as approximately 26.2 nm. XRD analysis also revealed that average crystallite size of Al_2_O_3_ increased from 8.8 nm to 26.2 nm, which confirms the deposition of Cu crystallites, with an increase in the mean thickness to ~17.4 nm. A similar XRD pattern was reported in literature [20,21], where the strongest peak of electroless deposited-Cu appeared at 2*θ* = 43°.

**Figure 3:**
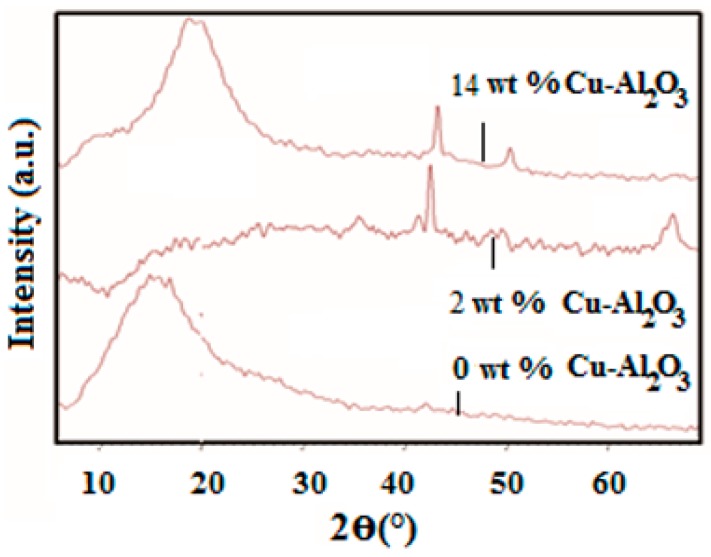
XRD patterns of neat polystyrene-block-poly(ethylene-ran-butylene)-block-polystyrene-graft-maleic anhydride (PS-*b*-(PE-*r*-B)-*b*-PS-*g*-MA) (0 wt % Cu-Al_2_O_3_) and composites with 2 wt % and 14 wt % Cu–Al_2_O_3_.

For determining the effect of Cu–Al_2_O_3_ filler in the host polymer matrix, XRD spectra of the polymer with 2 wt % and 14 wt % of Cu–Al_2_O_3_-loading were recorded (Figure 3). The XRD pattern of neat PS-*b*-(PE-*r*-B)-*b*-PS-*g*-MA shows a broad peak at 10°–27° and one relatively less intense peak at 48.9°, which confirms its amorphous structure. Upon 2 wt % Cu–Al_2_O_3_ loading in PS-*b*-(PE-*r*-B)-*b*-PS-*g*-MA, two peaks at 42.6° and 49.9° are observed. The peak at 42.6° is attributed to the crystalline nature of Cu, while another peak at 49.9° might represent a slight peak shift from 48.92° corresponding to amorphous phase of the polymer. At 14 wt % Cu-Al_2_O_3_ loading, three peaks at 2*θ* = 36.2°, 42.9° and 50.1° arise. The sharp peak observed at 42.9° is characteristic of metallic Cu inclusion supported by another peak at 50.1° and thus confirms the crystalline phase of the prepared composites.

### 3.2. Morphological Study of Cu–Al_2_O_3_ Filler and Block Copolymer Composites

**Figure 4:**
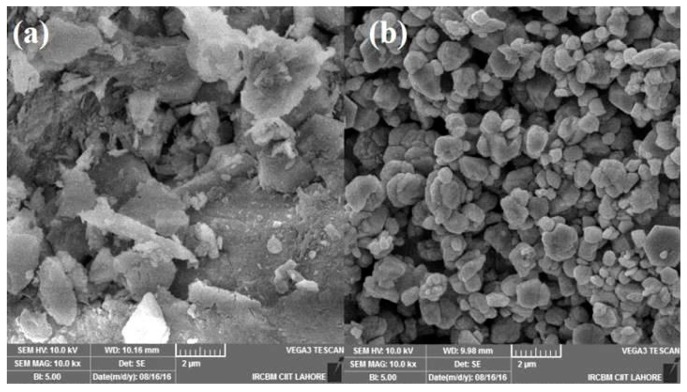

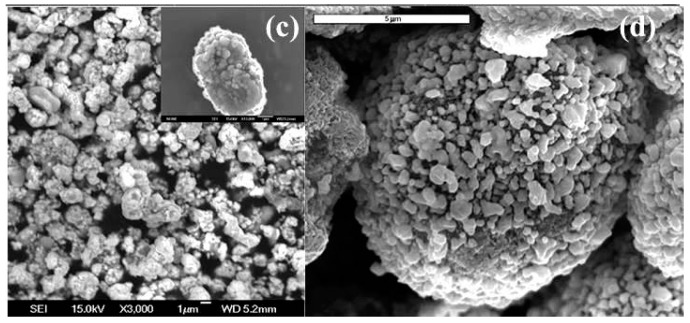
SEM micrographs of (**a**) pristine Al_2_O_3_ powder and Cu-coated Al_2_O_3_ (**b**) in this work; (**c**) in Wang et al. [25]; and (**d**) Ag coated onto polyimide [26].

SEM analysis was used to determine the surface morphology and crystalline structure of the materials. An SEM micrograph of pristine Al_2_O_3_ and Cu–Al_2_O_3_ powder are shown in Figure 4a,b, respectively, showing a uniform Cu coating on the alumina surface. The dispersion of the filler is improved as compared to pristine alumina powder. The Cu-coated Al_2_O_3_ particles exhibit fine-scale roughness, characteristic of metal coating [22,23]. Silvain and co-workers also deposited Cu onto submicron-sized Al_2_O_3_ particles [24]. Their work revealed uniform and fine coating of metallic Cu and increased average particle size of Al_2_O_3_ particles after Cu deposition. The SEM image of Cu–Al_2_O_3_ from Wang and co-workers [25] showed good similarity (Figure 4c). In comparison, Krupa and co-workers deposited Ag on polyimide particles (Figure 4d) [26]. In all these cases, the ELD-plating technique was used. The morphological properties looked similar and seem to be rather irrespective of the type of substrate.

**Figure 5:**
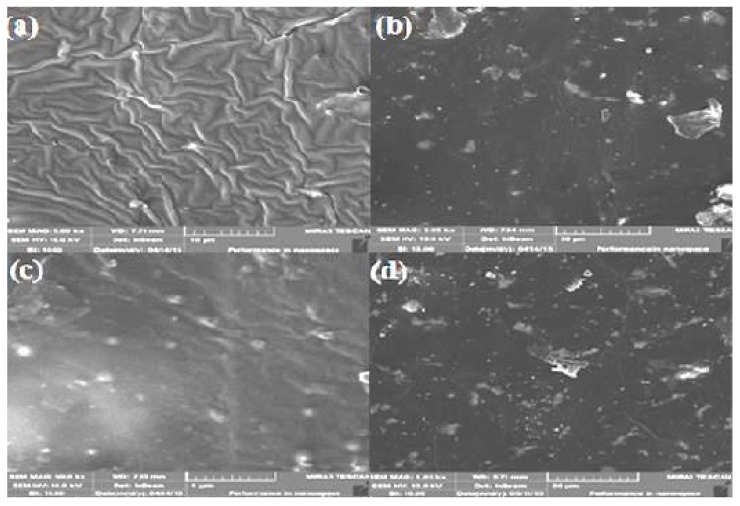

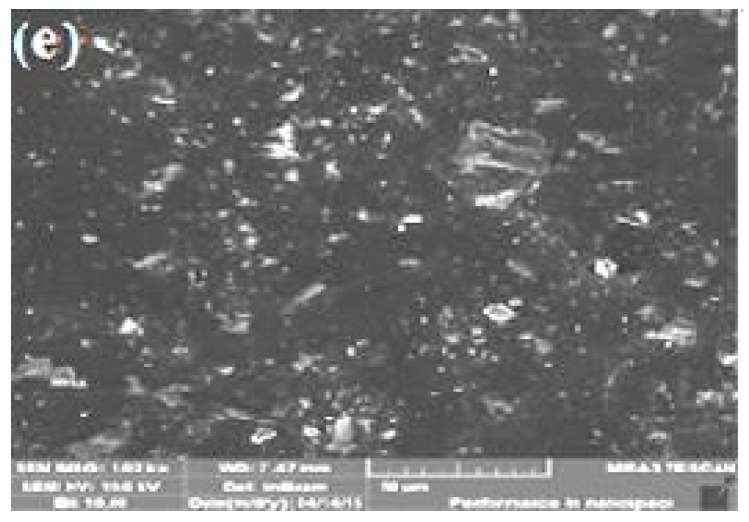
SEM micrographs of (**a**) PS-*b*-(PE-*r*-B)-*b*-PS-*g*-MA and composites with (**b**) 2; (**c**) 6; (**d**) 10; and (**e**) 14 wt % Cu–Al_2_O_3_.

The surface morphology of the host polymer PS-*b*-(PE-*r*-B)-*b*-PS-*g*-MA and the composite films with 2, 6, 10, and 14 wt % of Cu–Al_2_O_3_ are shown in Figure 5a–d. The incorporation of filler played a remarkable role on the morphology of the resultant composites. With the incorporation of lowest filler content (2 wt %), homogenous dispersion of filler is observed in both the composites predicting good filler–polymer interaction. At least 10 wt % filler is required to observe the initiation of particle-to-particle connectivities, which improve throughout the matrix when the filler content is further increased to 14 wt %. The comparison of Figure 5b,e illustrates the decreased interparticle distance. The shiny small areas in the SEM images resemble the presence of the metal coated on ceramic filler. Even smaller interparticle distance could be achieved with filler loadings higher than 14 wt %, but this compromises the mechanical performance considering the properties of ceramics. A clear transition in the particle shape and surface roughness takes place upon Cu metallization. The uniform growth of Cu crystallites on Al_2_O_3_ explains the change in morphology regarding particle distribution, which ultimately affects the mean coating thickness. At higher filler loading, agglomerates or islands of the filler particles are formed within the matrix material, which helps the smooth transfer of electrons. Individual nanosized filler particles are not distinctly visible in SEM micrographs because of this phenomenon [27].

### 3.3. EDS Analysis of Cu–Al_2_O_3_ Filler and Block Copolymer Composites

EDS was used to study the elemental composition of Cu–Al_2_O_3_ filler and Cu–Al_2_O_3_/PS-*b*-(PE-*r*-B)-*b*-PS-*g*-MA composites (Table 2). Cu, Al, and Pd were detected in the filler material. The high content of Cu (67.7%) followed by Al (30.4%) and Pd (1.9%) confirms the effective deposition of Cu–Al_2_O_3_ via the ELD process. Pd was present in small quantities, as it was used at a minor concentration for surface activation of the Al_2_O_3_ substrate.

### 3.4. Surface/Volume Resistivity and Electrical Conductivity of Block Copolymer Composites

The volume resistivity is the reciprocal of the electric conductivity. Measurement of the resistance across the materials’ surface, which is in contact with the electrodes, is termed surface resistivity (Ω/sq or Ω/□) [28,29], while electrical resistance through a cube of insulating material is considered as volume resistivity (Ω·cm). The host matrix polymers are usually non-conducting in nature and contain an insignificant number of charge carriers in free-state. Thus, the electrical properties of such matrix polymer composites almost exclusively depend on the selection of filler and its ability to form smooth conductive networks throughout the matrix [30,31,32,33,34,35]. The surface resistivity of Cu–Al_2_O_3_/PS-*b*-(PE-*r*-B)-*b*-PS-*g*-MA matrix composites with increasing filler loadings (2–14 wt %) were studied. The surface and volume resistivity of neat polymer was also analyzed to determine its electrical behavior as intrinsic or extrinsic conducting polymer matrix. Table 3 shows the surface resistivity, the volume resistivity, and the electrical conductivity. The values for PS-*b*-(PE-*r*-B)-*b*-PS-*g*-MA are 2.30 × 10^14^ Ω/□, 2.3 × 10^15^ Ω·cm, and 4.348 × 10^−16^ S/cm, respectively, which confirms that it cannot act as intrinsic conducting polymer; although bulky aromatic rings are present as pendants, the main chain is saturated, rendering an insulation material.

**Figure 6:**
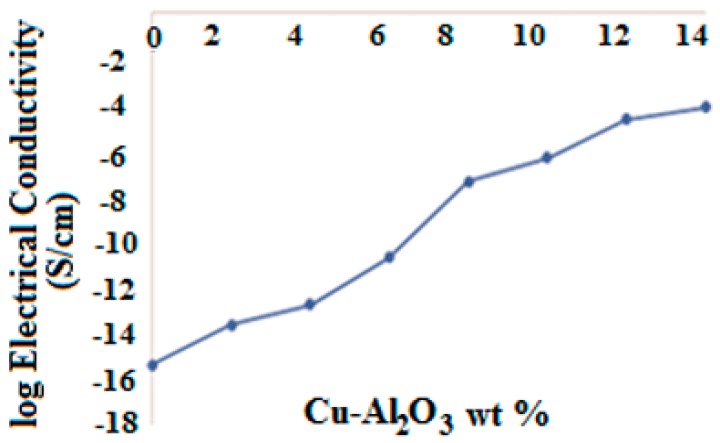
Electrical conductivity of Cu–Al_2_O_3_/PS-*b*-(PE-*r*-B)-*b*-PS-*g*-MA composites.

With the inclusion of small amounts of filler (2 wt %), the surface resistivity of the corresponding composite readily drops from insulating to antistatic region. The corresponding electrical conductivity increases to 2.381 × 10^14^ Ω·cm. This immediate shift from insulating to antistatic region might be attributed to the connection with unsaturated side chain substitutions like maleic anhydride and benzene groups, which essentially help to enhance particle-to-particle interaction [36]. By increasing the loading of Cu–Al_2_O_3_ filler in the polymer matrix from 4 to 12 wt %, the surface and volume resistivity drop from 5.8 × 10^9^ to 1.3 × 10^8^ Ω/□ and from 4.2 × 10^13^ to 4.5 × 10^4^ Ω·cm, respectively. This drop shifts the conductive properties of the material from the antistatic to the static dissipative region [37]. Upon further incorporation of filler (14 wt %), the surface resistivity drops drastically to 4.0 × 10^4^ Ω/□, while the volume resistivity and electric conductivity changed only substantially compared to 12 wt % filler loading. The gradual increment in conductivity with addition of 2–14 wt % filler is shown in Figure 6. It confirms network formation as suggested by the SEM results, showing the transition from an insulating to a semiconducting region.

**Figure 7:**
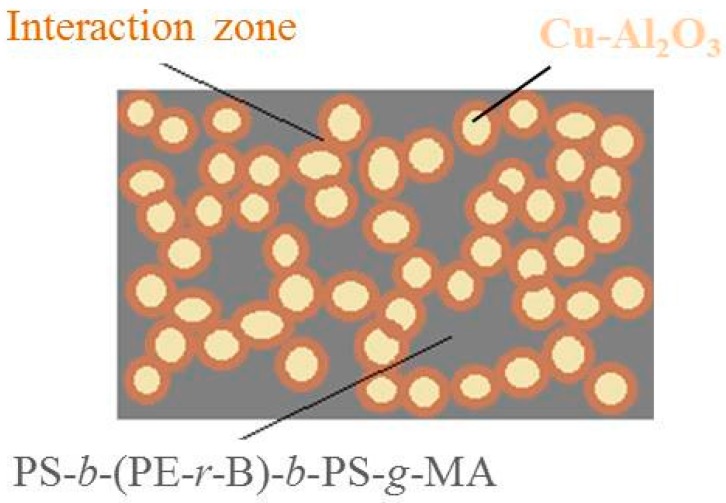
Schematic illustration of interaction between Cu–Al_2_O_3_ conductive filler and PS-*b*-(PE-*r*-B)-*b*-PS-*g*-MA polymer matrix responsible for electron transfer.

The electron transfer responsible for conductivity throughout the Cu–Al_2_O_3_/PS-*b*-(PE-*r*-B)-*b*-PS-*g*-MA matrix takes place when interaction zones between filler and matrix find connections (Figure 7), establishing a web [38,39,40,41]. Cu–Al_2_O_3_/PS-*b*-(PE-*r*-B)-*b*-PS-*g*-MA composites are cost-effective materials, as they showed enhanced electrical conductivity and they are easy to prepare compared to previously cited literature [42]. Beyond the critical concentration or percolation limit, there is no further significant increase in electrical conductivity even though more filler is contained in the composite material. Once the saturation point is attained, further increase in filler loading may only increase the sum of conductive networks and does not contribute in further conductivity increments. In contrast, shielding effectiveness may increase when higher filler loadings are used [43,44,45].

### 3.5. Mechanical Properties of Cu–Al_2_O_3_/PS-b-(PE-r-B)-b-PS-g-MA Composites

Mechanical performance of a polymer matrix composite can be influenced by the composition and interaction of filler and matrix materials used. Geometrical aspects, such as structure shape and size of reinforcement material, considerably affect the mechanical behavior of composites [46]. For the synthesis of structurally resilient composites, filler dispersion and declustering is a prerequisite. Thus, by critically controlling the volume fraction of filler, mechanical properties were measured to analyze the effect of filler inclusion and to prevent any deterioration in mechanical properties of composites [45]. The mechanical behavior of Cu–Al_2_O_3_/polymer composites was examined by calculating Young’s modulus, tensile strength, and % elongation at break of the composites with increasing filler loading (0–14 wt %).

#### 3.5.1. Young’s Modulus

**Figure 8:**
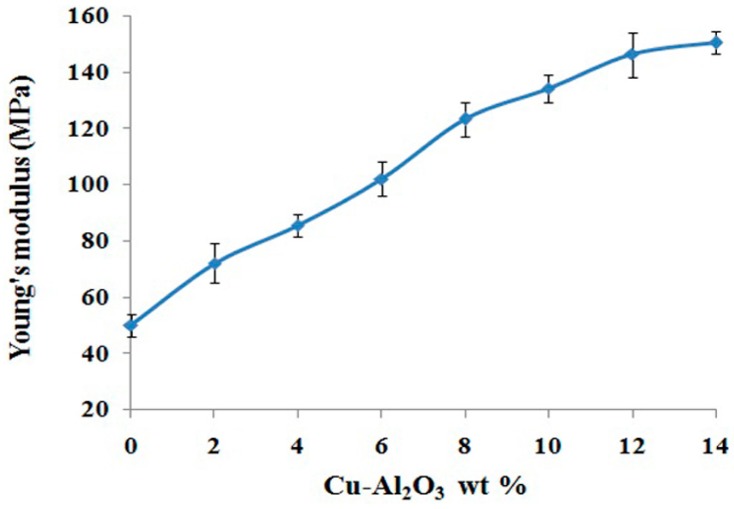
Young’s modulus of Cu–Al_2_O_3_/PS-*b*-(PE-*r*-B)-*b*-PS-*g*-MA composites.

Young’s modulus is a quantitative parameter for the stiffness determination of elastic materials. It is defined as the ratio of applied stress to the strain along the same axis. The applied stress should be in the range in which Hook’s law holds properly [47]. Young’s modulus of neat block copolymer is 50 ± 3 MPa, which increased to 150 ± 3 MPa (Figure 8) with the gradual addition of reinforcement material. This gradual and constant increase in Young’s modulus of composites with increased filler loading indicates enhancement in stiffness imparted by Al_2_O_3_.

#### 3.5.2. Tensile Strength

**Figure 9:**
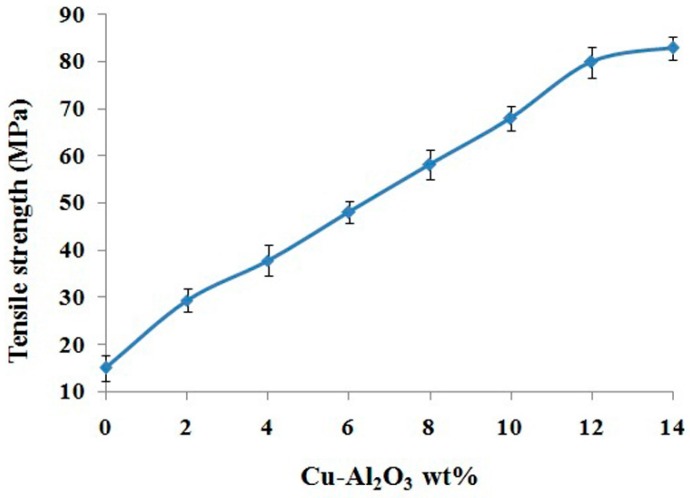
Tensile strength of Cu–Al_2_O_3_/PS-*b*-(PE-*r*-B)-*b*-PS-*g*-MA composites.

The maximum stress that a material can endure before failing or breaking is known as tensile strength [48]. The incorporation of Cu–Al_2_O_3_ in the polymer matrix increases the tensile strength of the resultant composites. At 14 wt % Cu–Al_2_O_3_ loading, the tensile strength of the composite reached 82 ± 3 MPa, as compared to 15 ± 3 MPa of the neat polymer. Figure 9 shows the gradual increase of tensile strength with filler loading. Tensile strength is strongly dependent upon interfacial adhesion/bonding between filler and matrix and is aided by uniform filler dispersion. Interfacial adhesion determines the strength of such composites. The results suggest good compatibility between particulate filler and polymer matrix and confirms active transfer of stress from matrix to particulate filler [49,50,51]. The PS-*b*-(PE-*r*-B)-*b*-PS-*g*-MA/Cu-Al_2_O_3_ composites offer good strength and mechanical resistance, compared to previously reported polymer/metal-coated polymers [26], polymer/carbon [42], polymer/ceramic [52], polymer/mineral ([53,54], ethylene–propylene–diene monomer rubber/Mg(OH)_2_) [55] and polymer/polymer composites [53], as illustrated in Table 4.

#### 3.5.3. Elongation at Break

**Figure 10:**
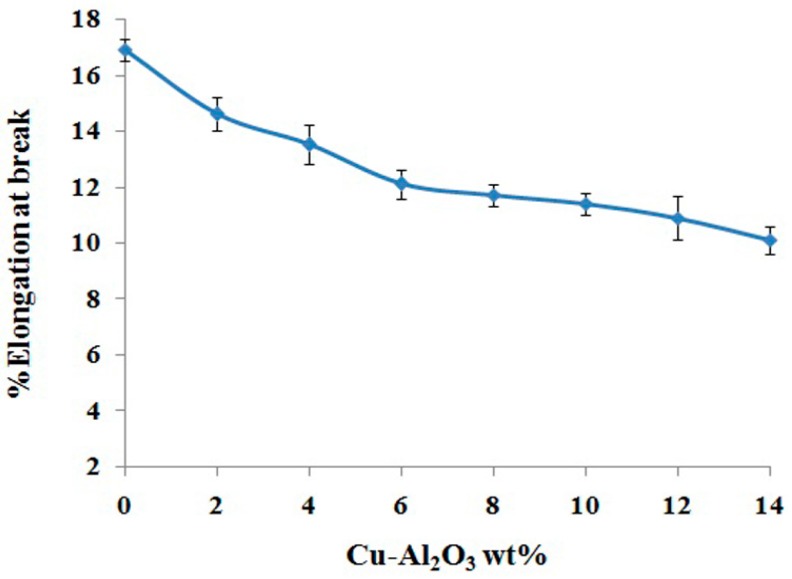
Elongation at break of Cu–Al_2_O_3_/PS-*b*-(PE-*r*-B)-*b*-PS-*g*-MA composites.

Elongation at break is a quantitative parameter for the ductility of the material. It is defined as the percentage of elongation of a material from zero stress to the breaking point of that material [56]. The elongation at break is also an indicator for determining the toughness of two phase materials [57]. The elongation at break calculated for PS-*b*-(PE-*r*-B)-*b*-PS-*g*-MA polymer was 16.9% ± 0.4%. Cu–Al_2_O_3_/PS-*b*-(PE-*r*-B)-*b*-PS-*g*-MA composites with increasing filler loadings (0%–14%) showed a gradual decrease from 16.9% to 10.1% (Figure 10). Polymers are ductile in nature while ceramics exhibit brittle behavior. Thus, the gradual increase in brittle behavior is due to the incorporation of the reinforcement material [58], and may arise from interstructural progression in which filler particles are dispersed in the interaggregate space [48]. At low filler loading, the matrix is not adequately reinforced. So, it could not withstand high load, and eventually failure happens at lower elongation. However, at higher filler loading, the matrix is increasingly reinforced and endures high load before the breaking point is reached. The reinforcement mechanism preludes that, at higher filler loading, the molecular mobility drops because of the formation of physical bonds among particles of filler and polymer molecule chains [43].

### 3.6. Thermal Characteristics of Block Copolymer Composites

#### 3.6.1. Thermogravimetric Analysis (TGA)

**Figure 11:**
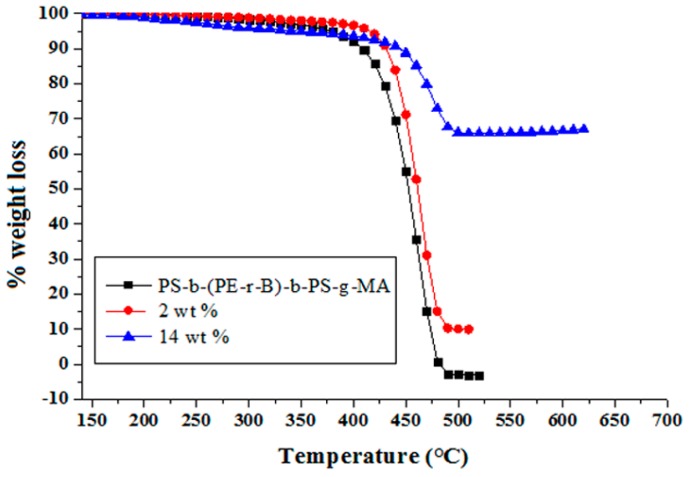
Thermogravimetric analysis (TGA) thermograms of neat PS-*b*-(PE-*r*-B)-*b*-PS-*g*-MA and composites with 2 wt % and 14 wt % Cu–Al_2_O_3_ loading.

**Figure 12:**
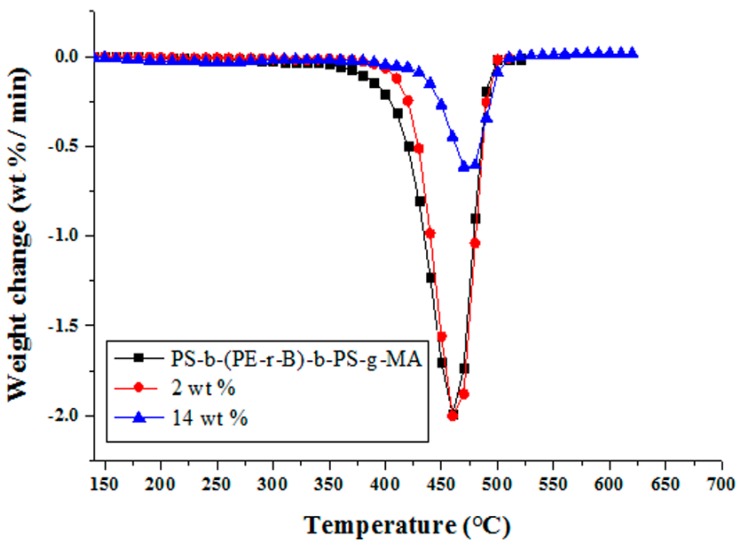
Derivative thermogravimetry (DTG) of neat PS-*b*-(PE-*r*-B)-*b*-PS-*g*-MA and composites with 2 wt % and 14 wt % Cu–Al_2_O_3_ loading.

TGA examines the thermal properties as the weight alteration upon heating during the phases of thermal breakdown. The thermal behavior determines the possible specific application fields of nanocomposites [59]. TGA thermograms of neat PS-*b*-(PE-*r*-B)-*b*-PS-*g*-MA and Cu–Al_2_O_3_ loaded composites from 0, 2, and 14 wt % are shown in Figure 11. A two-phase decomposition is observed for neat block copolymer. A slight dip at 250 °C indicates the presence of some residual low molecular weight compounds in the polymer. In the present conditions, the polymer remains stable up to 397 °C (8% weight loss). The second phase of decomposition starts at 397 °C (T_max_) and continues up to a final degradation temperature of 480 °C (99% weight loss at T_f_). With the inclusion of 2 wt % Cu–Al_2_O_3_, the thermal stability of the composite is improved, where T_max_ raises from 397 to 405 °C and T_f_ from 480 to 492 °C. At 14 wt % filler loading, T_max_ and T_f_ are respectively 30 °C and 9 °C higher compared to the neat polymer. At this point (T_f_ 489 °C), 67% residue is still left. Upon heating the polymer, the long chains break down into small fragments which might have interacted with Cu–Al_2_O_3_ particles and got trapped into filler particles difficult to be decomposed further, thus improving the thermal stability of the PS-*b*-(PE-*r*-B)-*b*-PS-*g*-MA composites [56]. Similar behavior was observed previously, where thermal stability was enhanced due to filler incorporation which hindered the segmental movement of polymer when intermingled with small chains of the host polymer [27,48]. Analogous degradation patterns are seen in the derivative thermogravimetry (DTG) curves of host polymer and its composites (Figure 12).

#### 3.6.2. Differential Scanning Calorimetry (DSC)

**Figure 13:**
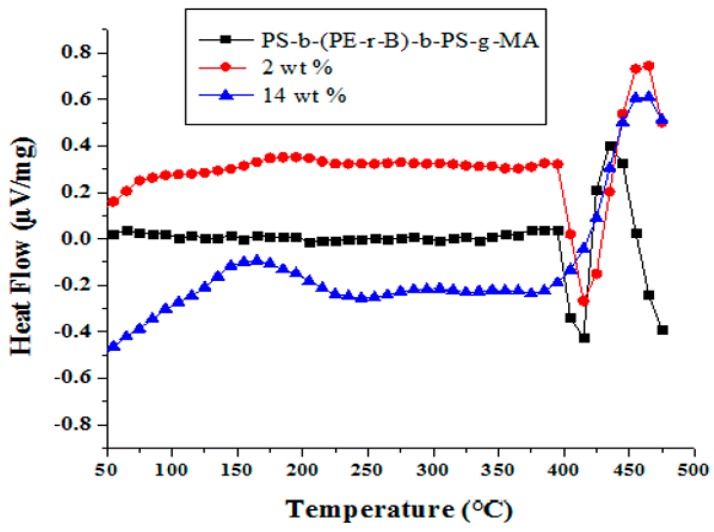
Differential scanning calorimetry (DSC) of neat PS-*b*-(PE-*r*-B)-*b*-PS-*g*-MA and composites with 2 wt % and 14 wt % Cu-Al_2_O_3_ loading.

DSC analysis provides the determination of the glass transition temperature (T_g_) of materials, the temperature at which a polymer transforms from a glassy to a rubbery state [60]. The DSC thermograms of neat PS-*b*-(PE-*r*-B)-*b*-PS-*g*-MA and the corresponding composites with 0, 2, and 14 wt % filler loading are shown in Figure 13. The stiffness of polymers is usually studied by T_g_ analysis. Stiff polymer chains with bulky, rigid side groups attached to the main chain imparts a high T_g_. It is known that at T_g_, polymer chains start to move. The results show that the incorporation of Cu–Al_2_O_3_ in the polymer matrix increases the T_g_ as the chains of PS-*b*-(PE-*r*-B)-*b*-PS-*g*-MA strongly adhere to the Cu–Al_2_O_3_ particles, which prevents free motion of polymer chains and hinders the segmental movement of chains [61,62].

## 4. Conclusions

In this study, nanocomposites were synthesized from the block copolymer polystyrene-*block*-poly(ethylene-*ran*-butylene)-*block*-polystyrene-graft-maleic anhydride (PS-*b*-(PE-*r*-B)-*b*-PS-*g*-MA) as the matrix and from a filler material, prepared by the electroless deposition (ELD) of Cu particles on alumina powder. The nanocomposite belongs to the class of inorganic–organic composites containing metal-coated ceramic reinforcement agent embedded in a thermoplastic polymer insulation, categorized as conductive polymer nanocomposites. The nanocomposites are easy to prepare, show enhanced electrical conductivity, improved thermal stability, and mechanical properties. The pronounced increment in electrical conductivity with increased filler ratio, up to 7.692 × 10^−5^ S/cm in the case of 14 wt % filler loading, indicates the formation of conductive networks within the prepared composites. A good interfacial adhesion between filler and matrix permits to improve the Young’s modulus and tensile strength at 14 wt % filler loading up to 159.475 MPa and 82.889 MPa, respectively. The composites also show improved thermal stability, while heat flow measurements via DSC show a higher glass transition temperature range with higher filler inclusion. XRD patterns indicate a more crystalline phase of the composites due to addition of metallic filler. SEM micrographs of the composites illustrate a uniform Cu deposition on Al_2_O_3_ and its homogeneous dispersion throughout polymer matrix when using the ELD technique. These results support the potential application of the prepared composites in electronic applications that require a prolonged shelf life, both in electronic semiconductors as in microelectronic packaging, EMI- and EDS-shielding materials, antistatic coatings for electronic, flexible IT devices, and others. Depending on the requirements of the applications, these materials may be used either in coatings or for standalone components.

## Figures and Tables

**Table 1:** Composition of electroless (EL) bath and conditions used for Cu plating.

Constituents of EL Bath	Chemicals	Amount (mmol)
Metal Salt	CuSO_4_·5H_2_O	64
Complexing Agent	KNaC_4_H_4_O_6_·4H_2_O	106
	Na_2_EDTA	54
	NaOH	350
Reducing Agent	HCHO	170
Stabilizer	CH_4_N_2_S	0.013
	C_2n_H_4n+2_O_n+1_	50 mL
Conditions in EL bath	Temperature	45–50 °C
	Time	30 min
	pH	12.0–12.5

**Table 2:** Elemental composition of Cu–Al_2_O_3_/PS-*b*-(PE-*r*-B)-*b*-PS-*g*-MA composites.

Cu–Al_2_O_3_ (wt %)	Atomic wt % of Elements			
	C	O	Al	Cu
0	95.3	4.7	–	–
2	85.1	4.3	1.3	9.3
14	79.2	3.5	0.4	16.9

**Table 3:** Surface/volume resistivity and electrical conductivity of Cu–Al_2_O_3_/PS-*b*-(PE-*r*-B)-*b*-PS-*g*-MA composites.

Cu–Al_2_O_3_ (wt %)	Surface Resistivity (Ω/□)	Volume Resistivity (Ω·cm)	Electrical Conductivity (S/cm)
0	2.3 × 10^14^	2.3 × 10^15^	4.35 × 10^−16^
2	4.2 × 10^10^	4.2 × 10^13^	2.38 × 10^−14^
4	5.8 × 10^9^	5.8 × 10^12^	1.72 × 10^−13^
6	5.1 × 10^9^	5.1 × 10^10^	1.96 × 10^−11^
8	2.3 × 10^8^	2.3 × 10^7^	4.35 × 10^−8^
10	2.1 × 10^8^	2.1 × 10^6^	4.76 × 10^−7^
12	1.3 × 10^8^	4.5 × 10^4^	2.22 × 10^−5^
14	4.0 × 10^4^	1.3 × 10^4^	7.69 × 10^−5^

**Table 4:** Comparison of the tensile strength of PS-*b*-(PE-*r*-B)-*b*-PS-*g*-MA/Cu-Al_2_O_3_ composites with previously reported data.

Composite Type	Tensile Strength (MPa)	Reference
PS-*b*-(PE-*r*-B)-*b*-PS-*g*-MA/Cu-Al_2_O_3_	82.9	Present research
Polyethylene/Ag-coated polyamide	2.7	[26]
Waterborne polyurethane/graphene	9.6	[42]
Polyurethane/silica	6.80	[52]
Polypropylene/CaCO_3_	29.7	[53]
Polypropylene/BaSO_4_	30.0	[54]
Ethylene–propylene–diene monomer rubber/MgOH_2_	9.6	[55]
Polypropylene/poly(methylmethacrylate)	29.5	[53]
